# Establishment, Implementation, Initial Outcomes, and Lessons Learned from Recent HIV Infection Surveillance Using a Rapid Test for Recent Infection Among Persons Newly Diagnosed With HIV in Thailand: Implementation Study

**DOI:** 10.2196/65124

**Published:** 2024-12-26

**Authors:** Kriengkrai Srithanaviboonchai, Thitipong Yingyong, Theerawit Tasaneeyapan, Supaporn Suparak, Supiya Jantaramanee, Benjawan Roudreo, Suvimon Tanpradech, Jarun Chuayen, Apiratee Kanphukiew, Thananda Naiwatanakul, Suchunya Aungkulanon, Michael Martin, Chunfu Yang, Bharat Parekh, Sanny Chen Northbrook

**Affiliations:** 1 Faculty of Medicine, Chiang Mai University, 110 Intavaroros road, Sriphum, Maung, Chiang Mai, 50200, Thailand, 66 818856142; 2 Research Institute for Health Sciences, Chiang Mai University, Chiang Mai, Thailand; 3 Department of Disease Control, Ministry of Public Health, Talard-Kwan, 88/21 Tiwanon Rd, Nonthaburi, Thailand, 66 02590300; 4 Division of Global HIV/TB, US Centers for Disease Control and Prevention, Nonthaburi, Thailand; 5 Department of Medical Sciences, Ministry of Public Health, Nonthaburi, Thailand; 6 Division of Global HIV/TB, US Centers for Disease Control and Prevention, Atlanta, GA, United States

**Keywords:** rapid test, surveillance, HIV, AIDS, diagnosis, Thailand, men who have sex with men, RITA, human immunodeficiency virus, acquired immune deficiency syndrome, transgender, recent infection testing algorithm

## Abstract

**Background:**

A recent infection testing algorithm (RITA) incorporating case surveillance (CS) with the rapid test for recent HIV infection (RTRI) was integrated into HIV testing services in Thailand as a small-scale pilot project in October 2020.

**Objective:**

We aimed to describe the lessons learned and initial outcomes obtained after the establishment of the nationwide recent HIV infection surveillance project from April through August 2022.

**Methods:**

We conducted desk reviews, developed a surveillance protocol and manual, selected sites, trained staff, implemented surveillance, and analyzed outcomes. Remnant blood specimens of consenting newly diagnosed individuals were tested using the Asanté HIV-1 Rapid Recency Assay. The duration of HIV infection was classified as RTRI-recent or RTRI-long-term. Individuals testing RTRI-recent with CD4 counts <200 cells/mm^3^ or those having opportunistic infections were classified as RITA-CS-long-term. Individuals testing RTRI-recent with CD4 counts >200 cells/mm^3^, no opportunistic infections, and not on antiretroviral treatment were classified as RITA-CS-recent.

**Results:**

Two hundred and one hospitals in 14 high-burden HIV provinces participated in the surveillance. Of these, 69 reported ≥1 HIV diagnosis during the surveillance period. Of 1053 newly diagnosed cases, 64 (6.1%) were classified as RITA-CS-recent. On multivariate analysis, self-reporting as transgender women (adjusted odds ratio [AOR] 7.41, 95% CI 1.59‐34.53) and men who have sex with men (AOR 2.59, 95% CI 1.02‐6.56) compared to heterosexual men, and students compared to office workers or employers (AOR 3.76, 95% CI 1.25‐11.35) were associated with RITA-CS-recent infection. The proper selection of surveillance sites, utilizing existing surveillance tools and systems, and conducting frequent follow-up and supervision visits were the most commonly cited lessons learned to inform the next surveillance phase.

**Conclusions:**

Recent HIV infection surveillance can provide an understanding of current epidemiologic trends to inform HIV prevention interventions to interrupt ongoing or recent HIV transmission. The key success factors of the HIV recent infection surveillance in Thailand include a thorough review of the existing HIV testing service delivery system, a streamlined workflow, strong laboratory and health services, and regular communication between sites and the Provincial Health Offices.

## Introduction

The early detection and treatment of newly infected persons is crucial for infectious disease control. In the case of HIV, people are more likely to pass on the virus to others due to high ongoing risk and unawareness of the infection [1-3]. The United States Centers for Disease Control and Prevention (US CDC) developed assays that can detect recent HIV infections, which included the less-sensitive enzyme immunoassay (EIA) [4], then the BED-Capture Enzyme Immunoassay [5], followed by the Limiting Antigen (LAg) Avidity EIA [6]. These laboratory tests have been widely used to estimate HIV-1 incidence in cross-sectional surveys. However, these laboratory-based assays must be performed centrally, and the test results are not available immediately. This limits the assays’ value in identifying those most likely to spread the infection.

Rapid testing can provide timely and useful information regarding recent HIV infections for surveillance purposes. Recent infection surveillance aids in locating hotspots of recent HIV infections, and when triangulated with data from other surveillance systems and program data, this may help identify populations and geographic areas where ongoing or recent transmission is occurring [7,8]. In 2013, the US CDC developed a novel rapid test [9], which later became commercially available as the Asanté HIV-1 Rapid Recency Assay (Sedia BioSciences, Beaverton, OR, USA). It can be used to simultaneously verify HIV infections and distinguish between long-term and recent HIV infections [10]. This lateral flow assay [11] was a format modification of the LAg Avidity EIA assay that distinguished long-term and recent HIV infections based on differential antibody binding at optimized LAg concentrations. The results can be interpreted both visually and with a strip reader. With this rapid test for recent HIV infection (RTRI), there is a potential to verify HIV diagnosis and detect recent infections on-site within minutes. To date, there are two commercially available point-of-care RTRI assays [12]. They are the Alere Ag/Ab Combo (Abbott Laboratory, Abbott Park, IL) that claims to detect acute infection before the development of HIV antibodies, and the Asante Recency Assay that detects recent infection post seroconversion. The Asanté HIV-1 Rapid Recency Assay has been validated in the laboratory as well as in the field and can detect more new infections due to a longer recency duration [10,13].

The TRACE initiative (Tracking with Recency Assays to Control the Epidemic) was launched in 2019. The surveillance was funded by the United States President’s Emergency Plan for AIDS Relief (PEPFAR) and implemented in several PEPFAR-supported countries [7]. Through TRACE, one of the recent infection testing algorithms (RITA) was implemented, which includes RTRI testing as an additional test in HIV testing services (HTS). The integration of RTRI into routine HTS offers an opportunity for program-based surveillance of persons with new HIV diagnoses in facility- and community-based testing sites [14].

Thailand joined the TRACE initiative in 2020. The project started as a demonstration project in Bangkok and expanded to include two additional provinces in 2021. During the first 2 years, RITA comprised RTRI and viral load (VL) testing for those diagnosed with RTRI-recent HIV; in combination with CS, this method was used to characterize HIV infections as recent or long-term [15]. In 2022, Thailand established the surveillance of recent HIV infections using RITA-CS. The use of VL testing as a proxy for antiretroviral treatment (ART) use was discontinued after the results from the demonstration project showed that the use of RITA-CS gave better results compared to RITA-VL [16]. Using CS to exclude or reclassify those who were previously diagnosed, those on ART, or those who had advanced HIV disease before or after RTRI testing improved the classification of recency status. In this paper, we describe the establishment of Thailand’s HIV-1 recent infection surveillance system using the RTRI test and CS, report the lessons learned, and initial results from April through August 2022.

## Methods

### Desk Review and Gathering of Relevant Information

A review of the findings and challenges/lessons from earlier TRACE projects conducted in Thailand and other nations was performed. The related service delivery and data management systems were also reviewed through a series of discussions with relevant organizations. These included the service delivery systems at HTS and ART clinics, the national algorithm of HIV diagnosis, logistics of CD4 and HIV VL laboratory testing, and current HIV-related data collection and data management system models.

### Development of Surveillance Protocols and Manuals

The essential components of the protocol were drafted based on protocols previously approved by the Thai Ethical Review Committee. The aim was to integrate recent infection surveillance into the existing nationwide surveillance system. The protocol’s overall draft, inclusion and exclusion criteria, service flow at the HTS, case record form, and data management system were handled by the Chiang Mai University Research Health Institute for Health Sciences (RIHES). It was the responsibility of the Thai Ministry of Public Health to draft the information regarding the supplies and logistics of the RTRI test kits, as well as the method of staff time and effort reimbursement. The RTRI laboratory training curriculum, procedure, and laboratory quality assurance/quality control protocol were the responsibility of the Thai National Institute of Health. The US CDC was responsible for drafting the informed consent and counseling process. The team meetings considered all inputs before developing the manual and staff training curriculum.

### Selection of Surveillance Sites

Provinces with >20% annual increase in new HIV diagnoses and new HIV diagnoses among men who have sex with men (MSM) and transgender women (TGW) from 2017 to 2020 were selected to participate. In Thailand, HTS are offered at public hospitals, private hospitals, private clinics, and certified community-based organizations. The majority of HIV testing occurs at public hospitals; therefore, as a first step, public hospitals within each selected province were invited to participate. Additional types of facilities may be involved in the next phase of the surveillance. Laboratory hubs were assigned based on the district of each participating hospital by the Provincial Health Office, except in Bangkok where each hospital had its own laboratory hub.

### Ethical Considerations

The underlying protocol was deemed a surveillance activity by the Thai Ministry of Public Health Ethics Committee and reviewed by the CDC; it was deemed not research and was conducted with applicable federal laws and CDC policies (eg, 45 C.F.R. part 46, 21 C.F.R. part 56; 42 U.S.C. §241(d); 5 U.S.C. §552 a; 44 U.S.C. §3501 et seq).

### Staff Training

There were two different types of training for the personnel involved in this project. The nurse counselor and laboratory staff were trained on the project overview, RTRI, service flow, data management, data use, and logistics of blood samples. The overall project training was couducted as a full day session, and hospitals were grouped into 6 groups for 6 separate trainings. Laboratory staff members were trained in RTRI laboratory techniques as well as associated internal quality control and external quality assessment procedures. This was a half-day training, and the sites were grouped into 3 groups for 3 separate trainings. Due to the COVID-19 situation at the time, the training was conducted online in March 2022.

### Implementation

Implementation began in April 2022 and ended in August 2022. The timing was determined by the budgetary requirements of the Thai government’s fiscal year and the need to allow sufficient time for data analysis and planning for the next phase of implementation. The project team went to all provincial sites to meet with the staff in person to discuss progress and problems with regard to the implementation and management of the project. The team also went to selected hospitals and laboratories that reported newly diagnosed HIV cases to gain firsthand information about the sites. These site visits served to monitor activities, address any problems delaying the commencement of activities, and address any queries that the sites had. The RIHES staff kept track of the data entered into the system and communicated with the sites regarding any missing data. Throughout this implementation phase, a series of online meetings with the provincial site coordinators were also held.

### Service Flow

#### Checking Eligibility Criteria

Using the national identification number, the HIV counselor checked whether the client had a previous HIV diagnosis or ART history in the National AIDS Program (NAP) database. Information on opportunistic infections (OIs) and CD4 count at the time of HIV diagnosis was retrieved from medical records once available and was used to improve the accuracy of the test as part of CS.

#### Verbal Informed Consent

Due to the limitations of coverage by trained staff, only clients who had HIV testing at HIV, tuberculosis, sexually transmitted infection, and antenatal clinics were covered by the project. During posttest HIV counseling, clients who tested positive for HIV following the national 3-test algorithm, those aged≥15 years old, those with no previous HIV diagnosis or ≤28 days of ART, those residing in selected provinces, and those able to communicate in Thai were asked for their consent to use remnant blood collected for HIV testing for RTRI. The RTRI result was only used for surveillance purposes and was not returned to the client.

#### RTRI Testing

The RTRI test was performed on the leftover blood samples for those RTRI-consented clients who tested HIV-positive. The RTRI results were classified as RTRI-recent (infection acquired in the past 12 months), RTRI-long-term (infection acquired more than 12 months ago), or RTRI-inconclusive and recorded in the project’s data management system by laboratory staff. We used CS as part of a RITA to identify late presenters. Individuals with a RTRI-recent result and CD4 counts <200 or OIs were reclassified as RITA-CS-long-term. All other RTRI-recent individuals were classified as RITA-CS-recent. To reduce the cost of the recency surveillance program, eligible blood samples from community hospitals that had a small number of clients were sent to the laboratory hubs for RTRI testing.

#### Data Collection

The team designed a 1-page RTRI case record form to collect individual client information: (1) demographics: age, sex, previously registered in the NAP database, nationality, residential postal code, marital status, and occupation; (2) HIV risk: key population, risk behaviors, risk venue, timing of the last risk behavior; and (3) RTRI result.

#### Data Management System

Alongside the data collection tools, a specific online data management system was created. Healthcare workers had two options for entering data: directly into the system or first writing it down on the paper form before entering it into the online system. Demographics and risk behaviors already entered into other data systems (hospital information system and laboratory data system) were transferred to the project’s data management system. Project staff checked the data in the system regularly for data inconsistencies and missing data before contacting the provincial health officer to assist with problem-solving.

### Data Analysis

Data were entered, cleaned, and analyzed using the Statistical Package for the Social Sciences (SPSS), version 22.0 (IBM Corp). Frequencies, percentages, and means were given in descriptive statistics when applicable. The *χ^2^* test was performed to compare categorical variables. The primary outcome was the proportion of RITA-CS-recent HIV infection among newly diagnosed individuals after excluding those with OI, CD4 <200 cells/mm^3^, previous HIV diagnoses, or ART history in the NAP database. The crude odds ratios (CORs) were calculated to determine risk factors for RITA-CS-recent HIV infection. All predictor variables with significant associations were included in multiple logistic regression after accounting for any potential multicollinearity. The variable “Geographical region of the HIV test,” was excluded from the multivariate analysis because it may not accurately represent the true residential regions and locations where the infections occurred of these highly mobile high-risk populations. The adjusted odds ratios (AORs) were calculated to determine independent risk factors for RITA-CS-recent HIV infection. We calculated 95% CIs for both simple and multivariate logistic regressions. For all analyses, a *P*-value of <.05 was regarded as statistically significant.

### Lessons Learned

A meeting was held with 26 co-investigators and participating staff and moderated by the Department of Disease Control after completing data collection to share lessons learned and inform planning and implementation of the next round of recency surveillance in 2023. A separate section of the meeting focused on lessons learned. The study team reviewed the process and presented the results, after which attendees were invited to share their thoughts and reach a consensus on whether specific items should be considered important lessons learned.

## Results

### Study Sites

There were 14 provincial sites participating in the surveillance. Only 1 province per health region was selected for the 13 health regions, except in region 7 that had 2 provinces. Altogether, 201 hospitals and 41 laboratory hubs volunteered to participate in recency surveillance. The variation in participating hospitals across provinces is due to the fact that invitation forms were distributed to each province, and participation was completely voluntary. The provincial teams, consisting of Provincial Health Officers and HIV coordinators from the hospitals, were responsible for determining the number and size of the hospitals involved (Table 1).

**Table 1.** Distribution and proportion of participating hospitals and corresponding laboratory hubs within each province by geographic region and health region, Thailand, April – August 2022.

Geographic region	Health region	Province	Participating hospitals, % (n/N)	No. of corresponding laboratory hubs
North	1	Chiang Rai	100 (18/18)	2
	2	Phitsanulok	22.2 (2/9)	2
	3	Kamphaeng Phet	100 (12/12)	3
Central	4	Nonthaburi	87.5 (7/8)	1
	5	Ratchburi	100 (11/11)	4
	6	Chon Buri	8.3 (1/12)	1
Northeast	7	Maha Sarakham	7.7 (1/13)	1
		Khon Kaen	7.7 (2/26)	2
	8	Udon Thani	4.5 (1/22)	1
	9	Buri Ram	100 (23/23)	2
	10	Mukdahan	53.8 (7/13)	1
South	11	Surat Thani	100 (21/21)	7
	12	Songkhla	100 (17/17)	4
Bangkok	13	Bangkok	83.9 (78/93)	11
Total			67.4 (201/298)	41

**Figure 1.**
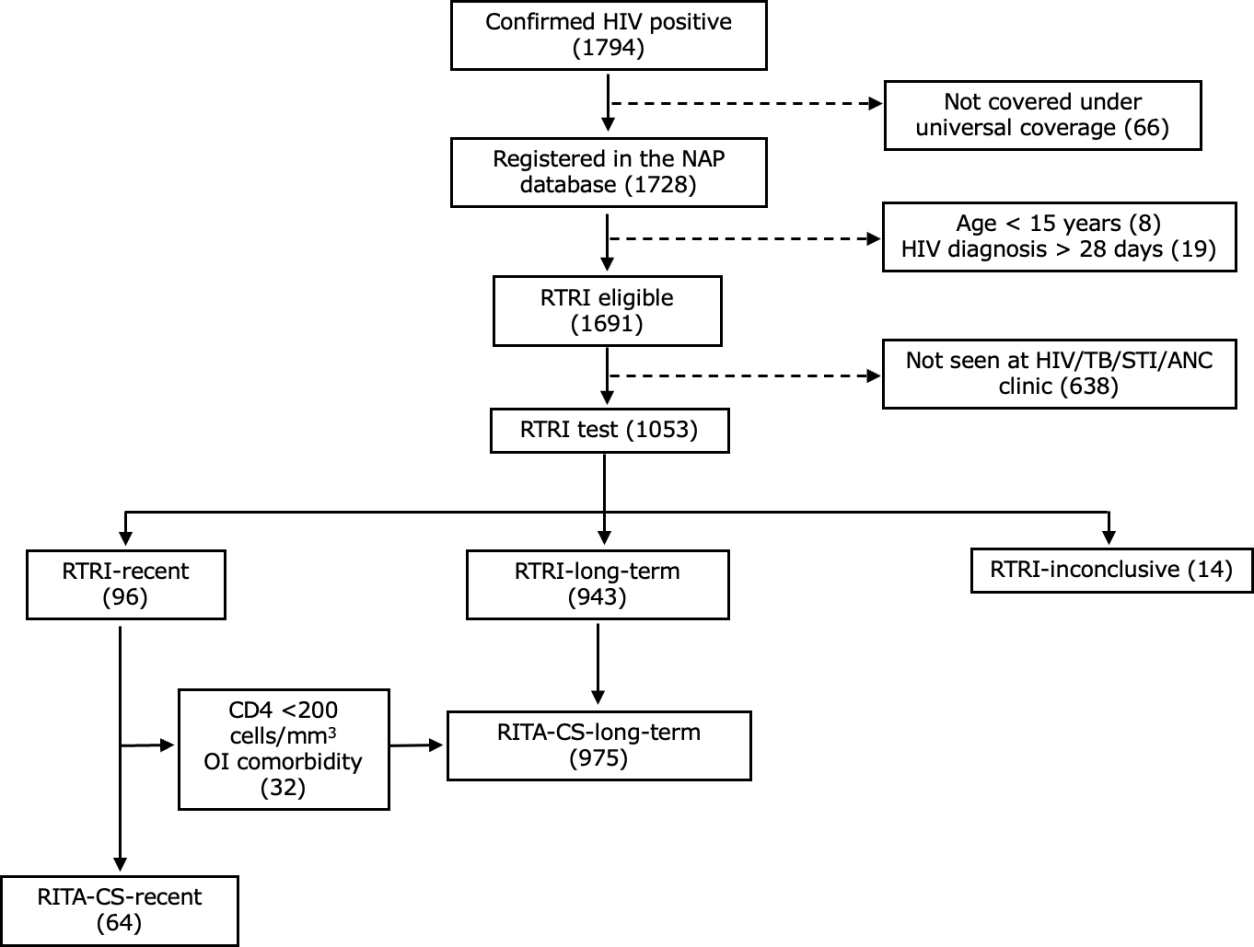
Flow diagram of reclassified RTRI-recent and reclassified RTRI-long-term HIV infections among newly diagnosed persons living with HIV in 14 selected provinces, Thailand, April – August 2022 (N=1784). ANC: antenatal clinic; OI: opportunistic infection; PLHIV: persons living with HIV; RITA: recent infection testing algorithm; RTRI: rapid test for recent infection; STI: sexually transmitted infection; TB: tuberculosis.

Of the 201 participating public hospitals, 69 reported at least 1 newly diagnosed HIV infection (range, 17‐260 newly diagnosed HIV individuals per facility) during the surveillance period. Among 1794 newly diagnosed HIV individuals, 1053 consented to the RTRI test. After excluding 14 inconclusive results, 96 (9.2%) were classified as RTRI-recent. Of these, 32 (33.3%) had an OI or CD4 counts <200 cells/mm^3^ at the time of HIV diagnosis and were reclassified as RITA-CS-long-term. A total of 64 (6.2%) were classified as RITA-CS-recent infections, and 975 (93.8%) were classified as RITA-CS-long-term infections (Figure 1).

Demographic comparisons between RITA-CS-recent and RITA-CS-long-term infections are shown in Table 2. Age, sex, marital status, geographic region of residence, and type of employment were statistically associated with the duration of HIV infection. Among RITA-CS-recent infections, the model categories were persons aged ≥20 years (82.8%), male (78.1%), MSM (48.4%), single (56.3%), residing in the Northeast region (46.9%), Thai (98.4%), and students (26.6%). Similar demographic characteristics were reported among RITA-CS-long-term infections, except for the type of employment with the majority reporting as laborers (36.0%).

**Table 2.** Demographic characteristics of RITA-CS-recent and RITA-CS-long term HIV infections among newly diagnosed persons living with HIV in 14 selected provinces, Thailand, April – August 2022 (n=1039).

Demographic characteristics	Total N=1039 N (%)	Duration of HIV infection status		
		RITA^a^-CS^b^-Recent n=64 N (%)	RITA-CS-Long-term n=975 N (%)	*P* value
Mean (SD) age=34.7±12.5 years				
15‐19 years	76 (7.3)	11 (17.2)	65 (6.7)	.005
≥20 years	963 (92.7)	53 (82.8)	910 (93.3)	
Sex				
Male	781 (75.2)	50 (78.1)	731 (75.0)	.572
Female	258 (24.8)	14 (21.9)	244 (25.0)	
Sex/sexuality				
Heterosexual	357 (34.4)	9 (14.1)	348 (35.7)	.004
Men who have sex with men	397 (38.2)	31 (48.4)	366 (37.5)	
Transgender	27 (2.6)	4 (6.3)	23 (2.4)	
Bisexual	24 (2.3)	3 (4.7)	21 (2.2)	
Other	168 (16.2)	10 (15.6)	158 (16.2)	
Missing	66 (6.4)	7 (10.9)	59 (6.1)	
Marital status				
Single	448 (43.1)	36 (56.3)	412 (42.3)	.032
Married	275 (26.5)	9 (14.1)	266 (27.3)	
Widow/Separate	56 (5.4)	1 (1.6)	55 (5.6)	
Missing	260 (25.0)	18 (28.1)	242 (24.8)	
Geographical region of residence				
Central	211 (20.3)	5 (7.8)	206 (21.1)	.012
North	92 (8.9)	5 (7.8)	87 (8.9)	
Northeast	319 (30.7)	30 (46.9)	289 (29.6)	
South	157 (15.1)	6 (9.4)	151 (15.5)	
Bangkok	260 (25.0)	18 (28.1)	242 (24.8)	
Nationality				
Thai	1,002 (96.4)	63 (98.4)	939 (96.3)	.373
Non-Thai	37 (3.6)	1 (1.6)	36 (3.7)	
Employment				
Unemployed	81 (7.8)	0 (0.0)	81 (7.8)	<.001
Office worker, government officer, employer	181 (17.4)	9 (14.1)	172 (17.6)	
Laborer	366 (35.2)	15 (23.4)	351 (36.0)	
Student	88 (8.5)	17 (26.6)	71 (7.3)	
Missing	323 (31.1)	23 (35.9)	300 (30.8)	

a RITA: recent infection testing algorithm.

b CS: case surveillance.

A significantly higher rate of RITA-CS-recent HIV infections was seen among those aged 15‐19 years (14.5%) compared to those aged ≥20 years (5.5%), MSM (7.8%) and TGW (14.8%) compared to heterosexual men (2.5%), those residing in the Northeast (9.4%) and Bangkok (6.9%) compared to the Central region (2.4%), and being a student (19.3%) compared to being an office worker/government officer or employer (5.0%). On multivariate analysis, self-reporting as MSM (AOR 2.59, 95% CI 1.02‐6.56), TGW (AOR 7.41, 95% CI 1.59‐34.53), and being a student (AOR 3.76, 95% CI 1.25‐11.35) were independent risk factors for RITA-CS-recent HIV infection after controlling for age and other variables (Table 3).

**Table 3.** Risk factors associated with RITA-CS-recent HIV infection among newly diagnosed persons living with HIV in 14 selected provinces, Thailand, April–August 2022 (n=1039).

Characteristics	RITA-CS-recent HIV infection (n=64)		
	% (n/N)	Crude odds ratio (95% CI)	Adjusted odds ratio (95% CI)
Age in years			
15‐19	14.5 (11/76)	2.91 (1.45‐5.83)^a^	1.53 (0.49‐4.75)
≥20	5.5 (53/963)	1	1
Sex			
Male	6.4 (50/781)	1.19 (0.65‐2.19)	a^b^
Female	5.4 (14/258)	1	
Sex/sexuality			
Heterosexual	2.5 (9/357)	1	1
Men who have sex with men	7.8 (31/397)	3.28 (1.54‐6.98)^a^	2.59 (1.02‐6.56)^a^
Transgender	14.8 (4/27)	6.73 (1.93‐23.50)^a^	7.41 (1.59‐34.53)^a^
Bisexual	12.5 (3/24)	5.52 (1.39‐21.93)^a^	4.43 (0.84‐23.47)
Marital status			
Single	8.0 (36/448)	4.81 (0.65‐35.75)	a^b^
Married	3.3 (9/275)	1.86 (0.23‐14.99)	
Widow/Separate	1.8 (1/56)	1	
Geographical region of residence			
Central	2.4 (5/211)	1	a^b^
North	5.4 (5/92)	2.34 (0.67‐8.39)	
Northeast	9.4 (30/319)	4.28 (1.63‐11.21)^a^	
South	3.8 (6/157)	1.64 (0.49‐5.46)	
Bangkok	6.9 (18/260)	3.06 (1.12‐8.40)^a^	
Nationality			
Thai	6.3 (63/1,002)	2.42 (0.33‐17.90)	a^b^
Non-Thai	2.7 (1/37)	1	
Employment			
Unemployed	0.0 (0/81)	n/a^c^	n/a^c^
Office worker, government officer, employer	5.0 (9/181)	1	1
Laborer	4.1 (15/366)	0.82 (0.6135‐1.90)	0.90 (0.35‐2.28)
Student	19.3 (17/88)	4.58 (1.95‐10.75)*	3.76 (1.25‐11.35)^a^

a Statistically significant (*P*<.05).

b a, not included in the analysis.

c n/a, not applicable.

### Lessons Learned

A number of lessons learned were identified to inform the next phase of recency surveillance: (a) exclude hospitals that do not report newly diagnosed HIV-positive infections in future plans; (b) utilize existing surveillance database, data management system, and dashboards in the next phase; (c) conduct frequent follow-up and supervision of sites to identify issues, challenges, and best practices that can be shared with others; (d) continue to provide laboratory training to ensure accuracy, reliability, and quality of RTRI testing; and (e) continue to monitor the accuracy of results across all laboratories and ensure robust quality control and external quality assurance systems are in place.

## Discussion

### Principal Findings

HIV recent infection surveillance combining CS and RTRI tests has been established and integrated into Thailand’s HIV surveillance system. Several factors contributed to the successful implementation of this project: (a) thorough review of existing related service delivery and data management systems, (b) development of a project workflow that was integrated into the existing HTS, (c) strong existing foundation of laboratory and health services, (d) early site visits to gather feedback for system improvement, (e) regular communication with the sites, and (f) development of a dashboard for the visualization and monitoring of key indicators such as the number of RTRI tests performed, proportion of RTRI-recent cases, and recent HIV infection across populations.

### Comparison With Previous Works

The use of RTRI alone can yield false recent results due to the potential misclassification of recent infection cases with advanced HIV disease, ART, or elite suppressors [17,18]. The World Health Organization and population-based HIV impact assessment surveys have combined recency assays with additional parameters, such as VL and/or checking for antiretroviral drugs in the blood, in lowering the false recent rate for HIV incidence estimates [15,19]. In Thailand, the initial demonstration project showed that combining CS data and RTRI testing (RITA-CS) was more accurate in classifying recent infection status than RTRI with VL testing (RITA-VL) (these data will be published in a separate report; data available on request). Our recency surveillance results indicated that those with AHD, when misclassified, could not be identified using RITA-VL due to their high VL but CS information helped improve their classification. The streamlined process of RTRI testing at HTS and CS data use has enabled Thailand to integrate the HIV recent infection surveillance into the routine surveillance system as reported here.

In addition to RTRI results, the history of the NAP database registration, OIs, and CD4 information were incorporated into the analysis. By excluding or reclassifying clients with evidence of long-term HIV infection (presence of OIs, CD4 counts <200 cells/mm^3^, prior HIV diagnosis, or ART history), our results showed a less biased proportion of recent infections among newly diagnosed persons living with HIV. The proportion of recent HIV infection dropped from 10.2% to 6.2% after reclassifying RTRI-recent clients with a history of HIV diagnosis, OIs, and CD4 counts <200 cells/mm^3^ as RITA-CS-long-term HIV infections. When determining RTRI-recent infections, this algorithm should be considered in countries with a strong CS, as it is more precise than utilizing RTRI results alone or with RITA-VL. Both RITA-CS and RTRI data were able to identify sex minority populations, such as TGW and MSM, as risk groups for recent HIV infection outside of students.

To increase the efficiency of the surveillance program, future plans should only include hospitals that are sufficient in size and have high-risk populations within their jurisdiction. To reduce the probability of misclassification, known cases of longstanding HIV infection should be screened out first using information from health records, both locally and nationally. These include clients who have previously registered for HIV, received an OI diagnosis, and had low CD4 counts. Additional information is needed to recommend whether Thailand should implement RTRI surveillance in HTS as one of its long-term country-wide HIV surveillance systems as there are potential biases when interpreting recency results from HTS programs [12]. However, this program-based surveillance can complement cross-sectional surveys and provide additional data for triangulation. As of now, the country considers RTRI a more convenient platform and plans to replace LAg EIA with RTRI for HIV surveillance among specific target groups, such as female sex workers and pregnant women, pending additional data and information.

Our study results align with the current trends in the HIV situation in Thailand. The Biological and Behavioral Surveillance data indicated a consistently high prevalence of HIV among MSM, TGW, and youth populations [20]. The NAP database revealed that MSM ranked first and TGW second among populations seeking HIV testing. Additionally, MSM and TGW have been the most frequently diagnosed groups with new HIV cases at HTS in recent years [21].

The Ministry of Public Health leadership plans to invite policymakers responsible for HIV prevention, testing, and care services to review and acknowledge the study results. To adapt Thailand’s existing programs in response to the high prevalence of recent HIV infections among MSM, TGW, and youth students, it is crucial to implement targeted strategies that address the specific needs of each population. The following are potential adaptations to be considered: enhanced outreach and education programs promoting HIV prevention, including condom distribution, preexposure prophylaxis, and postexposure prophylaxis; promoting early HIV testing and expanding HIV self-testing through targeted channels for specific populations; strengthening linkages and referral systems for HIV testing and treatment; and establishing comprehensive health services that enable healthcare providers to serve key populations and youth. These services should address both sexual and mental health concerns related to HIV and sexually transmitted infections, with tailored treatment options for high-risk groups.

Klock et al used the Asanté HIV-1 Rapid Recency Assay for the estimation of incidence in a cross-sectional survey [22]. Although the estimate was lower than the LAg-EIA estimate or MAA-C estimate (0.92 vs 1.29 or 1.26, respectively), the 95% CI overlap suggests that the estimates are not different. It is to be noted that the CDC (developer of the assay) does not recommend the use of the Asante assay for estimating the incidence considering its visual interpretation, which can be subjective. However, the assay works well with adequate training and in the context of program-based recent infection surveillance, as used in Thailand and in several other studies [8,10,13,14].

Galiwango et al, using the data from 45 seroconverting samples, concluded that Asante has suboptimal sensitivity [23]. In fact, as pointed out in the 2022 World Health Organization technical guidance [12], “sensitivity” is not a relevant term in the context of recency assays. Seroconversion panels are used to estimate the mean duration of recent infection of a recency assay, not to determine sensitivity. This mean duration of recent infection applies to the population, not to individuals. There is a distribution around the mean and individual recency period can vary from 2‐3 months to 8‐9 months (all within 1 year) depending on when they transition from recent to long-term status. This is true not only for Asante but also for other recency assays. Therefore, at the individual level, it is appropriate to use the phrase, as being infected within the past 12 months.

### Limitations

There are several limitations of this analysis. First, as participation was voluntary and not all HTS sites within large hospitals or hospitals within a selected province participated in this surveillance round, HIV diagnoses identified during the surveillance period may not be representative of the catchment area or province. Second, only 1 or 2 sites were selected to represent each province, and the results may not be generalizable to the entire province. Third, we gathered self-reported information on demographics. Fourth, individuals who frequently retest may be more likely to receive a recent infection test result than individuals who rarely test due to self-stigma, low perception of HIV risk, and other factors (eg, long travel distance to clinic or low HIV knowledge). Finally, we were not able to retest newly diagnosed PLHIV with an RTRI-inconclusive result and excluded them from the analysis.

### Conclusion

Through recent infection surveillance, we identified newly diagnosed individuals who self-reported as MSM or TGW, and students to be more likely to have acquired the infection recently at the time of HIV testing. Key factors contributing to the establishment and implementation of the HIV recent infection surveillance in Thailand include a thorough review of the existing HTS service delivery system, a streamlined workflow, a strong foundation of laboratory and health services, and regular communication between sites and the Provincial Health Offices. When triangulated with other programs and surveillance data sources, RTRI-recency data may provide a better understanding of current epidemiologic trends. Targeted interventions to prevent new infections among youth, TGW, and MSM may help interrupt ongoing transmission in Thailand in near real-time.

## Abbreviations

AOR: adjusted odds ratio
ART: antiretroviral treatment
COR: crude odds ratio
CS: case surveillance
EIA: enzyme immunoassay
HTS: HIV testing services
LAg: limiting antigen
MSM: men who have sex with men
NAP: National AIDS Program
OI: opportunistic infection
PEPFAR: President’s Emergency Plan for AIDS Relief
RIHES: Research Health Institute for Health Sciences
RITA: recent infection testing algorithm
RTRI: rapid test for recent HIV infection
TGW: transgender women
TRACE: Tracking with Recency Assays to Control the Epidemic
US CDC: United States Centers for Disease Control and Prevention
VL: viral load

## References

[1] Ambrosioni J, Junier T, Delhumeau C (2012). Impact of highly active antiretroviral therapy on the molecular epidemiology of newly diagnosed HIV infections. AIDS.

[2] Paraskevis D, Nikolopoulos G, Tsiara C (2011). HIV-1 outbreak among injecting drug users in Greece, 2011: a preliminary report. Euro Surveill.

[3] Wawer MJ, Gray RH, Sewankambo NK (2005). Rates of HIV-1 transmission per coital act, by stage of HIV-1 infection, in Rakai, Uganda. J Infect Dis.

[4] Janssen RS, Satten GA, Stramer SL (1998). New testing strategy to detect early HIV-1 infection for use in incidence estimates and for clinical and prevention purposes. JAMA.

[5] Parekh BS, Kennedy MS, Dobbs T (2002). Quantitative detection of increasing HIV type 1 antibodies after seroconversion: a simple assay for detecting recent HIV infection and estimating incidence. AIDS Res Hum Retroviruses.

[6] Duong YT, Qiu M, De AK (2012). Detection of recent HIV-1 infection using a new limiting-antigen avidity assay: potential for HIV-1 incidence estimates and avidity maturation studies. PLoS ONE.

[7] Kim AA, Behel S, Northbrook S, Parekh BS (2019). Tracking with recency assays to control the epidemic: real-time HIV surveillance and public health response. AIDS.

[8] Telford CT, Tessema Z, Msukwa M, Arons MM, Theu J, Bangara FF (2022). Geospatial transmission hotspots of recent HIV infection. MMWR.

[9] Granade TC, Nguyen S, Kuehl DS, Parekh BS (2013). Development of a novel rapid HIV test for simultaneous detection of recent or long-term HIV type 1 infection using a single testing device. AIDS Res Hum Retroviruses.

[10] Yufenyuy EL, Detorio M, Dobbs T (2022). Performance evaluation of the Asante Rapid Recency Assay for verification of HIV diagnosis and detection of recent HIV-1 infections: implications for epidemic control. PLOS Glob Public Health.

[11] Sadeghi P, Sohrabi H, Hejazi M (2021). Lateral flow assays (LFA) as an alternative medical diagnosis method for detection of virus species: the intertwine of nanotechnology with sensing strategies. Trends Anal Chem.

[12] (2022). Using recency assays for HIV surveillance: 2022 technical guidance.

[13] Agyemang EA, Kim AA, Dobbs T (2022). Performance of a novel rapid test for recent HIV infection among newly-diagnosed pregnant adolescent girls and young women in four high-HIV-prevalence districts-Malawi, 2017-2018. PLoS ONE.

[14] Rwibasira GN, Malamba SS, Musengimana G (2021). Recent infections among individuals with a new HIV diagnosis in Rwanda, 2018-2020. PLoS ONE.

[15] Kassanjee R, Pilcher CD, Busch MP (2016). Viral load criteria and threshold optimization to improve HIV incidence assay characteristics. AIDS.

[16] Suraratdecha C, MacKellar D, Hlophe T (2022). Evaluation of community-based, mobile HIV-care, peer-delivered linkage case management in Manzini Region, Eswatini. Int J Environ Res Public Health.

[17] Hladik W, Olara D, Mermin J (2012). Effect of CD4+ T cell count and antiretroviral treatment on two serological HIV incidence assays. AIDS Res Hum Retroviruses.

[18] Laeyendecker O, Rothman RE, Henson C (2008). The effect of viral suppression on cross-sectional incidence testing in the johns hopkins hospital emergency department. J Acquir Immune Defic Syndr.

[19] Voetsch AC, Duong YT, Stupp P (2021). HIV-1 recent infection testing algorithm with antiretroviral drug detection to improve accuracy of incidence estimates. J Acquir Immune Defic Syndr.

[20] Department of Disease Control (2024). HIV info hub. https://hivhub.ddc.moph.go.th/epidemic.php.

[21] National Health Security Office (2024). NAP: web report. http://napdl.nhso.go.th/NAPWebReport/main_care.jsp#.

[22] Klock E, Wilson E, Fernandez RE (2021). Validation of population-level HIV-1 incidence estimation by cross-sectional incidence assays in the HPTN 071 (PopART) trial. J Int AIDS Soc.

[23] Galiwango RM, Ssuuna C, Kaleebu P (2021). Short communication: validation of the Asante HIV-1 rapid recency assay for detection of recent HIV-1 infections in Uganda. AIDS Res Hum Retroviruses.

